# POLICY AND ECONOMIC INFLUENCES ON RETIREMENT, FINANCIAL LITERACY, AND ECONOMIC SECURITY IN JAMAICA

**DOI:** 10.1093/geroni/igz038.2150

**Published:** 2019-11-08

**Authors:** Julian G McKoy Davis

**Affiliations:** Mona Ageing & Wellness Centre, Kingston, Jamaica

## Abstract

The changing pension landscape, as well as the accompanying privatization, marketization and individualization of the pension planning process, has resulted in inadequate and risky investment practices. Jamaica, like many other countries in the international community, has been engaged in pension reform. The main issues in the current dispensation of pension reform are in relation to pension adequacy to mitigate against longevity risk, low levels of pension coverage; with the aforementioned leading to the need to increase the number of persons contributing to the pension pool as well as possibly an increase in the value of pension contributions to account for inflation and investment risks. This paper describes the current legal and regulatory framework on pension reform in Jamaica within the context of population ageing and the National Financial Inclusion Strategy that targets persons who were previously underserved by the domestic financial system. Policy and programmatic recommendations are will be provided.

